# The Gaggle: An open-source software system for integrating bioinformatics software and data sources

**DOI:** 10.1186/1471-2105-7-176

**Published:** 2006-03-28

**Authors:** Paul T Shannon, David J Reiss, Richard Bonneau, Nitin S Baliga

**Affiliations:** 1 Institute for Systems Biology, 1441 N 34^th ^Street, Seattle, WA 98103, USA; 2 Department of Biology, New York University, 100 Washington Square E, New York, NY 10003, USA

## Abstract

**Background:**

Systems biologists work with many kinds of data, from many different sources, using a variety of software tools. Each of these tools typically excels at one type of analysis, such as of microarrays, of metabolic networks and of predicted protein structure. A crucial challenge is to combine the capabilities of these (and other forthcoming) data resources and tools to create a data exploration and analysis environment that does justice to the variety and complexity of systems biology data sets. A solution to this problem should recognize that data types, formats and software in this high throughput age of biology are constantly changing.

**Results:**

In this paper we describe the Gaggle -a simple, open-source Java software environment that helps to solve the problem of software and database integration. Guided by the classic software engineering strategy of *separation of concerns *and a policy of *semantic flexibility*, it integrates existing popular programs and web resources into a user-friendly, easily-extended environment.

We demonstrate that four simple data types (names, matrices, networks, and associative arrays) are sufficient to bring together diverse databases and software. We highlight some capabilities of the Gaggle with an exploration of *Helicobacter pylori *pathogenesis genes, in which we identify a putative ricin-like protein -a discovery made possible by simultaneous data exploration using a wide range of publicly available data and a variety of popular bioinformatics software tools.

**Conclusion:**

We have integrated diverse databases (for example, KEGG, BioCyc, String) and software (Cytoscape, DataMatrixViewer, R statistical environment, and TIGR Microarray Expression Viewer). Through this loose coupling of diverse software and databases the Gaggle enables simultaneous exploration of experimental data (mRNA and protein abundance, protein-protein and protein-DNA interactions), functional associations (operon, chromosomal proximity, phylogenetic pattern), metabolic pathways (KEGG) and Pubmed abstracts (STRING web resource), creating an exploratory environment useful to 'web browser and spreadsheet biologists', to statistically savvy computational biologists, and those in between. The Gaggle uses Java RMI and Java Web Start technologies and can be found at .

## Background

The practice of systems biology seeks to comprehend the complexity of organisms, or their subsystems, by combining many different kinds of data (mRNA and protein levels, protein-protein and protein-DNA interactions, protein modifications, biochemistry, etc.) to create predictive models [1]. In current practice, as biologists explore their data, they typically create manual, *ad hoc *connections among software tools and databases, cutting and pasting queries, creating temporary files, running web searches, and taking notes. This strategy does not scale well, and so, in response, several software projects have arisen to offer computer-assisted data and software integration. Notable among these are ToolBus [2], Taverna [3], caCore [4], each of which uses semantic mapping to ensure that entities in one environment are appropriately related to entities in another. While it is appealing in the abstract, this approach can be quite costly, which may explain why, despite many person-years of engineering effort, these projects have not yet been widely adopted in the biological community.

## Implementation

### The Gaggle: an overview

The Gaggle [5] uses a minimalist approach to integrate data and software. It is written in Java and uses standard Java libraries. It is simple to install, and easy to update; new data sources and software tools can be added with minimal implementation costs. A small server program (the '*Gaggle Boss*') provides communication among analysis and display programs (the '*geese'*) which are modest adaptations of existing (or novel) bioinformatics and computational biology programs and web resources. The Boss and the geese all run as separate programs on the user's desktop computer, communicating with each other, at the user's behest, by passing simple messages.

In the Gaggle, ***semantic flexibility ***[6] – the notion that "word meanings are not ... fixed and unchanging, but tend to vary according to the context of their use" – is seen as a *solution *to the complications of data integration, rather than as a problem that must be solved before integration can begin. Four data types (names, matrices, networks, and associative arrays), distilled into a semantically simple form, are passed between the geese, whereupon they take on richer meaning in the context of each goose. For example, the gene name "*HP0352" *identifies (1) a flagellar motor switch protein in three KEGG [7] pathway maps, (2) a node in a Cytoscape [8] association network, (3) a row in a matrix to a microarray data viewer, and (4) a set of PubMed abstracts to a literature search tool. The biological semantics attached to the gene name in each of these environments are rich, significant, and though overlapping, somewhat *different*. But in the Gaggle's approach to software and data integration, no formal mapping and no explicit integration are needed. It suffices to simply pass the gene name (accompanied by the organism name, which is required by some geese) to each environment, where in each case a different web of meanings is invoked.

The Gaggle does not, however, preclude the use of applications and data repositories which are built upon, and offer the benefits of, careful semantic mapping. This is demonstrated by the KEGG goose, which submits queries to and retrieves results from KEGG. All carefully curated semantic mappings of gene name to metabolic pathways, biochemical reactions, cellular structures, DNA sequences, protein functions, and orthology groups in KEGG are obtained by merely passing gene names to this goose. As systems biology matures, we predict that many more such semantically rigorous resources will become available, and that they too will be easy to add to the Gaggle using this same approach. Similarly, large scale efforts such as SBML (Systems Biology Markup Language,[9], BioMoby [10] and BioPAX (a collaborative effort to create a data exchange format for biological pathway data)[11] will continue to be complementary to the Gaggle. However, we are also confident that, given the heterogeneity of systems biology data, it is unlikely that a single unifying language or unifying scheme will emerge. Valuable work will continue to be done in more or less restricted domains and semantic flexibility will always be required to integrate them.

### The Gaggle in action: A simple introductory example

**Figure 1:**
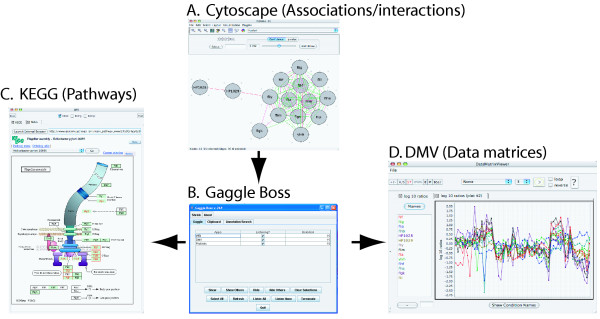
**A simple introductory example for use of Gaggle**. A set of genes (circular nodes with edges represents associations/interactions) selected in **Cytoscape **(**A**) are broadcasted to the **Gaggle Boss **(**B**). The Gaggle Boss re-routes the broadcast to a Java web browser connected to KEGG (**C**), further exploration wherein localizes *H. pylori *proteins to relevant subunits in the flagellar apparatus map. A second goose that receives the broadcast is the DMV (**D**). A plot function therein provides mRNA levels of the 15 *H. pylori *genes in 57 experimental conditions.

In a simple, prototypical use of the Gaggle, genes of interest are first selected in some program – perhaps nodes in a Cytoscape network. Next, by pressing the "Broadcast" button, the selected gene names are sent to other geese: the KEGG goose and a microarray data viewer goose. The KEGG goose will respond by displaying a list of pathways and structures in which those genes are implicated, and the microarray data viewer will plot the experimental data for those genes (Fig 1).

Thus, a single mouse click performs all operations which would otherwise require: (1) writing down, copying or exporting the names of the selected Cytoscape nodes to a file; (2) browsing to the 'Search Objects in KEGG Pathways' web page, and typing in, pasting, or otherwise loading the gene names from the file, making sure to prefix each name with the appropriate organism code; and finally (3) plotting the microarray data, again requiring the list of gene names to be typed, loaded, or pasted into the microarray data viewing program's 'select' function. In our experience, in absence of the Gaggle, such data exploration involving more than a few genes can be tedious and error-prone. With the Gaggle, even with a large number of genes, such exploration can be fast and dependable and can easily include a wide range of tools and data sources, all of which respond to a single mouse-click.

### Gaggle data types

There are four data types used in the Gaggle, and broadcast at the user's request from program to program. They are all implemented as Java classes, and they are all free of explicit biological semantics. They are: a list of names, a matrix, a network, and an associative array (a collection of name/value pairs). These four are sufficient to capture all the kinds of data used in systems biology. Instances of these data types are transmitted in *serialized *form using Java RMI within the Gaggle. Name lists and associative arrays are standard Java classes; Network and DataMatrix are Gaggle-specific, and are documented at the Gaggle website. Some straightforward translation of these types into 'native' types is usually required when adapting a new program to the Gaggle.

### The Gaggle boss

The Gaggle always has a **Boss**. This is a standalone Java program (usually, but see Section 4 below for an alternate approach) that relays messages between the programs in the Gaggle. The Boss's graphical user interface (GUI) displays the names of the currently registered programs, and provides the user with some basic controls: to hide or reveal particular programs, and to specify whether they are to accept or ignore messages. The Boss can, in addition, be given extra capabilities, which are added via a plugin mechanism. In the example below (see Section 3), we use a species-specific annotation search capability which appears as a tab in the Boss's GUI.

### Current Geese

When any Java program has been adapted to run in the Gaggle, we call it a '*goose*', and we say that it has been '*gaggled*'. This is a relatively simple operation: to gaggle a program requires only that it implement a dozen or so new programming calls (see software design and engineering below). Current geese in the Gaggle include:

**1**. **DMV: **the DataMatrixViewer, for navigating and selecting from experiments (microarray, ChIP-chip, proteomics), and for displaying and plotting their numerical data (Johnson et al, in preparation).

**2**. **Cytoscape, with assorted plugins, **for viewing protein-protein interactions, protein-DNA interactions, association networks, biclusters discovered by the cMonkey algorithm (see below; Reiss et al., submitted)[8].

**3**. **TIGR's microarray expression viewer (TMev): **a popular tool for microarray analysis [12].

**4**. **The R Goose: **using Java-to-R translation classes provided by RoSuDa the R Goose provides full access to the R statistical programming language and its many packages, including BioConductor [13].

**5**. **Simple Bioinformatics Web Browser: **which provides easy access to web-based bioinformatics resources, e.g., KEGG, EMBL's STRING, BioCyc [8,14,15].

### Starting the Gaggle

The Gaggle is often launched using Java Web Start [16] – a standard Java technology for launching programs from a single click in a web browser. The Gaggle Boss and any number of geese may be set up, for example, as links on a laboratory's web page, perhaps including shared laboratory data; any scientist can then launch the programs with a few mouse clicks. With Java Web Start, each goose is downloaded from the web server the first time it is run; it then runs locally on one's desktop computer like a standard installed program. On every subsequent launch of the program, a fresh version is downloaded only if the program has been updated on the web server. Web Start therefore simplifies distribution and maintenance of the Gaggle, and of shared data. Java Web Start, however, is not with a requirement of the Gaggle; traditional installation and update procedures work fine as well.

### Supporting other programming languages in the Gaggle

Though Java is an excellent general purpose language, it is not the right tool for every job and many bioinformatics tools are written in other languages. ***R ***[17,18], for example, is the language of choice for statistics, C++ is preferred for applications in which speed is essential, and Python and Perl are scripting languages popular in the bioinformatics community. Three strategies are available for accommodating these and other languages: cross-language interoperability (using the Java Native Interface, JNI [19]), JVM-rehosting (i.e., Jython as a rehosted Python[20]), and web services (in which Simple Object Access Protocol [SOAP] [21] provides remote, language-neutral access to programs written in other languages).

We employ the first strategy (JNI) in the R goose. The second strategy, JVM-rehosting, allows Python programs to join the Gaggle; we use the resulting Jython geese for prototyping and debugging. Jython geese and the R goose are excellent tools for exploratory data analyses that require scripting.

Perl and C++ are not yet available directly within the Gaggle. In order to use code written in these languages, a few possibilities exist: either JNI 'glue' code must be written; the code must be made available as through SOAP as a web service; or the code must appear on the web behind a CGI interface.

### Software design and engineering

The Gaggle's design is based upon the classic software engineering strategies of *separation of concerns *[22], and *parsimony*, from which we derived these specific prescriptions: (1) use the fewest possible software elements, (2) keep each maximally ignorant of all others, (3) avoid biological semantics, (4) use mainstream programming languages, and only one such language if possible, (5) avoid operating systems dependencies, (6) make sure that existing popular software and data formats are supported, (7) place a priority on ease of installation and update. These principles led us to choose the general purpose programming language Java, which has additional noteworthy features, including portability across operating systems, a simple and robust inter-process communication (RMI, remote method invocation), and the means (JNI) to call programs written in other languages.

Every program which runs in the Gaggle is a *separate, stand-alone *program. A Gaggle Boss (also typically, but not necessarily, a stand-alone program) is always started first. It provides a graphical interface to monitor and control the geese, and using RMI, the communications infrastructure. Every goose, at startup, registers itself with the current boss.

We use the traditional Java *interface *mechanism to specify both the extent to which each goose is aware of the boss and also the capabilities necessary for a program to become a full member of the goose. A Java interface defines a *type*, without specifying how that type is implemented. This common programming strategy allows for the separation of *what *an object must do, from *how *it does it. In the Gaggle, for example, every gaggled program must provide a *handleNameList *method (which is called when a bunch of gene names are broadcast to it), but the actual *implementation *of this method will differ with every Goose. These are presented below, followed by detailed explanations of some typical implementations of key methods in these interfaces. A full, compilable, and annotated listing of a minimal, functioning Goose will be found in the supplement.

Java RMI is the linchpin of the Gaggle. This standard Java component is a very sophisticated and robust technology for inter-process communication; fortunately, it is also very simple to use. It works like this: after an initial lookup to obtain a reference to the remote object (a remote program) one program can subsequently call methods on that remote program just as if it were a local object. In the Gaggle, we use RMI to broadcast data, and for housekeeping chores (i.e., to hide, show, or terminate specific geese, to get and set their names). The four Gaggle data types (see above) are all *serializable*, which means that Java RMI can send 'across the wire' to the remote program, marshalling and demarshalling the data at each end. The four data types are defined as Java classes, but all of them may be written to and read from disk in various formats, of which plain text and xml currently dominate. Within a running gaggle, however, all of the data exists strictly as Java objects.

The Gaggle defines two simple class interfaces (Boss and Goose), as well as the four data types. A Goose is an existing Java program adapted to run in the Gaggle; the adapation may be a plugin, a derived class, or an object added to the existing Java program. Only the methods listed below need to be implemented by every goose. Since these methods – especially in the prototyping stage – can be stubs (empty functions), the simplest adaptation of a program to the Gaggle can be very simple indeed, as illustrated below:

Goose.java

public interface Goose extends Remote

void connectToGaggle ();

void handleNameList (String species, String [] names);

void handleMatrix (DataMatrix matrix) throws RemoteException;

void handleMap (String species, String dataTitle, HashMap hashMap);

void handleCluster (String species, String clusterName, String [] rowNames, String [] columnNames);

void handleNetwork (String species, Network network);

String getName ();

void setName (String newName);

void doHide ();

void doShow ();

void doExit () ;

...

}

Let's examine three representative Goose methods (again, see the supplement for a fully documented simple goose).

### ConnectToGaggle

This method looks up the address of the boss, registers itself with the boss, and receives a unique name in response. (The goose has a preferred name, but if that name is already in use, the boss will make sure the returned name is unique.) Henceforth the goose and the boss each have a reference to each other, and can communicate any of the messages specified in the other's interface. The crucial lines of code in this method are

boss = (Boss) Naming.lookup ("rmi://localhost/gaggle");

myGaggleName = boss.register ((Goose) this);

### handleNameList

Perhaps the most used Goose method. When one goose broadcasts a list of names to another, this is the method which executes in the receiving goose. The full signature of the method is

handleNameList (String species, String [] names)

where the '*names' *denote entities (often genes) in the organism named in the '*species' *variable. In a typical implementation, i.e., in a network viewing program, this method would highlight all of the nodes whose names appear in the variable *names*.

### DoHide

This is an example of a Gaggle housekeeping method. The boss calls this method on the goose, without additional arguments. The goose that receives the message typically responds by calling *mainframe.setVisible (false) *on its outermost JFrame.

Boss.java

public interface Boss extends Remote {

void String register (Goose goose);

void broadcast (String sourceGoose, String species, String [] names);

void broadcast (String sourceGoose, DataMatrix matrix);

void broadcast (String sourceGoose, String species, HashMap hashMap);

void broadcast (String sourceGoose, String species, String clusterName, String [] rowNames, String [] columnNames;

void broadcast (String sourceGoose, String species, Network network)

...

}

In addition to the "***boss.register***" call shown above, a goose will make calls to one or more of the Boss *broadcast *methods. The prototypical example here is that broadcast method which is overloaded for sending a list of names. The full signature is

broadcast (String sourceGooseName, String species, String [] names);

Here, ***sourceGooseName ***identifies the goose which initiated the broadcast, ***names ***are typically of genes or proteins of interest, and ***species ***identifies the organism from which the gene or protein names come. One benefit for requiring the name of the goose initiating the broadcast (***sourceGooseName***) is that this allows the boss to avoid broadcasting back to the goose from which the broadcast originated.

When the boss receives this message, the boss will rebroadcast the message to the other geese in the gaggle, calling ***handleNameList (species, names) ***on all listening geese (see Goose.java, above). And thus we come full circle, broadcasting a list of names from the source goose, to the boss, and then to one or more destination geese. This sequence of events, of course, is usually initiated by the biologist clicking a 'broadcast' button in a gaggled bioinformatics program in which some number of genes or proteins have been selected.

Please not that, in the current implementation, the Boss is a standalone program, but it could easily be re-implemented as a part of some *other *program. This might be attractive to a biologist with a favorite bioinformatics program to which they wish to add Gaggle capabilities. (A Cytoscape plugin, for example, could implement the Boss interface, and recreate the Boss user interface as a dedicated Cytoscape panel, thereby creating a Cytoscape-centric Gaggle.)

### Scalability of the Gaggle

#### (i) Adding new programs and web resources

Many programs and web sites can be added to the Gaggle quite easily. In every case, the ratio of software development time to bioinformatics benefit must be assessed; the benefits will often be worth the effort. Furthermore, although gaggling a program usually (not always) requires access to the source code, a lot of molecular biology software is open source, and a lot of it is written in Java. The R Goose and TIGR MeV are prime examples: these are popular and powerful software packages developed entirely independent of the Gaggle; each required only about a week of programmer time to adapt to the Gaggle.

Specifically, adding a Java program to the Gaggle is straightforward:

a. If the source code is available

b. If the data structures to be broadcast or received in the prospective goose are roughly compatible with the four data types used in the Gaggle (name lists, networks, matrices, associative arrays).

As for any program, adding a website to the gaggle also runs the gamut, from easy to complicated to onerous. The difficulty goes up when Javascript is used, if logins are required, if results are available only after substantial delay, and (especially) if the website undergoes frequent revision.

A third kind of prospective goose is a non-Java program. This will

a. require an experienced Java programmer familiar with Java JNI, and

b. separate development and compilation on each target operating system.

Please note, however, that it is not unusual to find a Java JNI bridge already created for other programming languages and environments: R, Python, Prolog, and Matlab, to mention a few.

#### (ii) Performance

With regard to run-time scalability: since the Gaggle's purpose is first and foremost to facilitate *interactive *exploration of multiple data types, and since human-computer interaction with desktop software is not computationally intensive, even inexpensive computers can easily keep up with the typing and mouse operations of any user, and with the performance requirements of most individual software programs. In typical use, the Gaggle user moves at a pace measured in seconds between the various gaggled programs, with only one program in the foreground at a time; all other gaggled programs are relatively inactive in the background, perhaps even swapped out into virtual memory. Thus, the normal use of the Gaggle scales very nicely: there is no practical limit to the number of relatively inactive programs which can reside in the background.

In the worst case scenario, if a sophisticated Gaggle user should broadcast large matrices or networks to several different analytical programs at once, and if all of these are running on the desktop computer, a scaling problem might result. But please note that this is not a problem with the Gaggle: this is the familiar problem of running too many simultaneous, computationally intensive tasks on a small computer. This could be considered a limitation of the Gaggle only if it promotes a style of work that might lead the biologist to attempt these multiple tasks at once when they otherwise would not have done so. If such situations were to arise and computationally intensive tasks start to swamp the desktop computer these tasks could be reconfigured to run on a server. In the Gaggle's case, this familiar solution can be implemented easily through a lightweight goose, from which the biologist can monitor and control the remote computationally-intensive task.

## Results and discussion

Using the Gaggle we have integrated diverse databases (for example, KEGG, BioCyc, String) and software (for example, Cytoscape, DataMatrixViewer, R statistical environment, and TIGR Microarray Expression Viewer). This loose coupling of diverse software and databases enables simultaneous exploration of experimental data (mRNA and protein abundance, protein-protein and protein-DNA interactions), functional associations (operon, chromosomal proximity, phylogenetic pattern), metabolic pathways (KEGG) and Pubmed abstracts (STRING web resource). More importantly, the researcher can craft queries to explore these rich resources without any software constraints. This is best demonstrated through the case study provided below.

### A case study: Exploring pathogenesis in *Helicobacter pylori*

We turn now to a demonstration of the Gaggle, where we explore diverse sets of publicly available data for *Helicobacter pylori *using a variety of bioinformatics software tools. The choice of an organism outside of our general expertise (which is the systems biology of archaeal organisms) is intentional; it demonstrates how data integration via software interoperability in the Gaggle can reveal, even to the relatively inexpert researcher, insights previously hidden from view. We conclude the example by making several discoveries including identification of a protein functionally associated with flagellar biosynthesis proteins with a predicted three-dimensional structure match to ebulin, a ricin-like toxin.

The steps listed below reflect a possible thought process of a biologist and indicate logic behind his/her actions. Moreover, this exercise exemplifies typical systems biology data exploration and analysis in the Gaggle. Specifically, as in any real-world systems biology data exploration, this workflow contains frequent dead ends, reiteration of the same or similar analyses with different parameters, and exploration of additional data to support new findings. The H. pylori demo is available on the Gaggle website.

**Figure 2:**
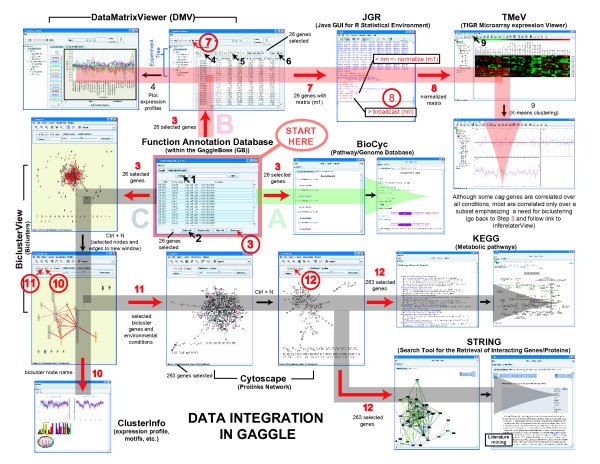
**Workflow used in Gaggle for exploration of H. pylori pathogenesis (see text for details)**. The exploration begins with the Gaggle Boss (GB). All steps (mouse clicks) are indicated by arrows alongside numbers (both in black and red font) that correspond to sequence of actions. Black numbers indicate actions within a goose; red arrows and numbers (enclosed in red circles) indicate "Broadcast" actions with corresponding red numbers (not enclosed in circles) indicating transmission of data from one goose to another (implicitly through the GB). The three watermark arrows in (A) green, (B) red and (C) grey provide sequence and paths of exploratory routes.

In this example we make use of diverse types of data archived in different locations around the world: Chromosome maps at BioCyc [15]; a local copy of publicly available mRNA data from Stanford microarray database [23]; functional associations from Prolinks [24];protein-protein interactions [25]; a local copy of a gene/protein annotation file from TIGR; metabolic pathways at KEGG [7] in Japan; and all Pubmed abstracts, and protein and DNA sequences through STRING [14] in Germany. The example also demonstrates the power of the Gaggle platform in enabling software interoperability, by including the DataMatrix Viewer (DMV) for exploring microarray data; JGR goose for statistical analysis using the 'R' package; and TMeV for clustering analysis of microarray data. More importantly, it showcases how broadcasting no more than 4 messages types through the Gaggle boss can catalyze seamless integration of all of these data and software (screenshots shown in Fig 2).

#### *Step-by-step demonstration of Gaggle exploration of H. pylori systems biology data *(see accompanying Fig 2 for details)

The goal of this analysis was to identify genes functionally associated with cytotoxin-associated genes of *H. pylori*

Step 1. We searched for the term "cag" (short for 'cytotoxin-associated gene') in the "Annotation Search" tab of the Gaggle Boss, identifying 26 genes encoded in the *H. pylori *genome (The annotations were obtained from the Comprehensive Microbial Resource at TIGR .

Step 2. All records (Total 26 genes) retrieved in Step 1 were selected within the annotation tab.

Step 3. The selected genes were broadcast to BioCyc [26], DMV (Johnson et al, submitted) and BiclusterView (see below, Reiss et al, submitted) (by selecting appropriate check boxes in the Gaggle Boss main panel). Taking Route A, records matching the 26 genes were retrieved through a web query against the BioCyc database for *Helicobacter pylori*. Upon following links for individual records we learned more about genome organization of these genes (for example *cag19 *seems to be in an operon along with *cag21*, *cag20 *and *cag18*) and that all *cag *genes are encoded contiguously within an approximately 40 Kbp pathogenicity island.

Step 4. After reviewing basic information for genomic organization of *cag *genes we took Route B to investigate relationships among them by exploring expression profiles in microarray data downloaded from the Stanford Microarray Database [27]. Route B begins with analysis of mRNA profiles for the 26 *cag *genes in the DMV; these genes were selected through a broadcast described in Step 3. By clicking the "Plot Profiles" button we visualized the expression profiles for all *cag *genes within the DMV. This indicated that the relationship among their expression profiles is complex and requires clustering analysis for proper evaluation.

Step 5. Using the "Create New Matrix" feature of DMV a sub-matrix of the 26 *cag *genes were created within the DMV.

Step 6. Subsequently all genes within this new sub-matrix were selected using the "Select All" feature.

Step 7. To ensure uniformity in the expression data across conditions we decided to normalize the data (variance = 1 and mean = 0). The selected sub-matrix was broadcast to JGR. This matrix was received as R-object *m1 *in JGR. The data were then normalized to matrix *nm *using an R function (*normalize*).

Step 8. The normalized sub-matrix (*nm*) was broadcast (using command "*broadcast (nm)*" within JGR) to TMeV [12] for further analysis.

Step 9. Expression profiles were clustered using the k-means algorithm (k = 5, Euclidean correlation metric) within TMeV. Upon viewing the 5 k-means clusters it was evident that whereas some *cag *genes, such as *cag1*, *cag20, cag21 *and *cag26*, had correlated profiles over almost all conditions, others (for example, *cag2*, *cag11*, *cag12*, *cag15, cag24 *and *cag25*) were correlated only under a subset of conditions. This elevated the importance of using a more sophisticated clustering procedure such as *cMonkey *(Reiss et al, submitted), which identifies putative groups of genes co-regulated over a subset of conditions (*biclusters*) by simultaneously analyzing expression data, functional and/or physical associations, and *de novo *detected cis-regulatory motifs (Reiss et al, submitted). We have developed a simple Cytoscape plug-in goose for filtering and exploring these biclusters (called BiclusterView), along with a PDF file viewer goose (called ClusterInfo) for viewing specific cluster information such as detected cis-regulatory motifs.

Step 10. Taking Route C, 19 biclusters containing pathogenicity genes (selected within the BiclusterView through a broadcast action described in Step 3) were sent to a new BiclusterView window (Ctrl + N). 12 of these biclusters shared metabolic processes and/or contained genes encoding physically interacting proteins, suggesting that these biclusters are functionally related. Properties of all biclusters (expression correlation, conserved motifs etc.) were further explored by broadcasting them using the "broadcast node names" feature to the ClusterInfo application. All biclusters were found to be of high quality and some contained a motif implicated in pH regulation (Reiss etal, submitted).

Step 11. To further explore functional associations among the pathogenicity genes, all genes contained within the 19 biclusters were broadcast to the Cytoscape view of the *H. pylori *Prolinks [24] Network using the "Broadcast genes and conditions" feature within the BiclusterView Control panel. All selected nodes (263 genes) within the selected Prolinks subnetwork were sent to a new window (Ctrl + N) along with associated edges. Altogether 203 genes within this network were interconnected through the following relationships: 85 gene cluster edges, 13 gene fusion edges, 99 gene neighbor edges, 83 phylogenetic profile edges and 53 protein-protein interaction edges [24,28]. Viewing functions for these 263 genes in the GB annotation tab revealed that many complex functions are associated with the cag gene blicusters (Additional file 1) elevating the need for further analysis with KEGG and STRING (below).

Step 12. Finally, we broadcast all genes in the Prolinks sub-network to KEGG [7] and STRING [14] to explore metabolic pathways represented in these biclusters as well as literature containing co-occurrence of two or more genes in these 19 biclusters. Altogether ~25 pathways with three or more enzyme matches were retrieved from KEGG (Additional file 2) and ~927 publications were retrieved through STRING. Within the abstract of these publications were co-occurrences of two or more genes from the 19 biclusters (or their orthologs in other organisms). Given the large number of papers that were retrieved, we subsequently conducted repeated searches in STRING by broadcasting fewer numbers of genes at a time. In the section below we provide a synthesis of our findings.

#### Summary of findings (Fig 3)

Using the Gaggle we were able to tease out from a complicated landscape of 6399 putative associations and physical interactions among 1539 genes, 57 microarray conditions, 246 gene biclusters, and nearly 79 KEGG pathways a far more easily comprehensible picture from which to gain biological insight. Specifically, in 12 steps we identified several previously known and also unknown relationships that could serve as tangible leads for future experimental investigation of *H. pylori *pathogenesis.

Among the pathways containing the filtered set of 263 genes (Additional file 1) was an over-representation of major processes that have been previously implicated in aspects of pathogenesis such as peptidoglycan biosynthesis [29], lipopolysaccharide biosynthesis [30], flagellar biosynthesis [31], Type IV secretion [32] (Additional file 2, Fig 3). Also present was an overrepresentation of enzymes for aa-tRNA synthesis, reductive carboxylate cycle, pyruvate metabolism, lysine biosynthesis, oxidative phosphoorylation and glycolysis/gluconeogenesis. Categorizing the 263 genes into these various pathways helped explore putative roles for proteins of unknown function (Additional file 1).

**Figure 3:**
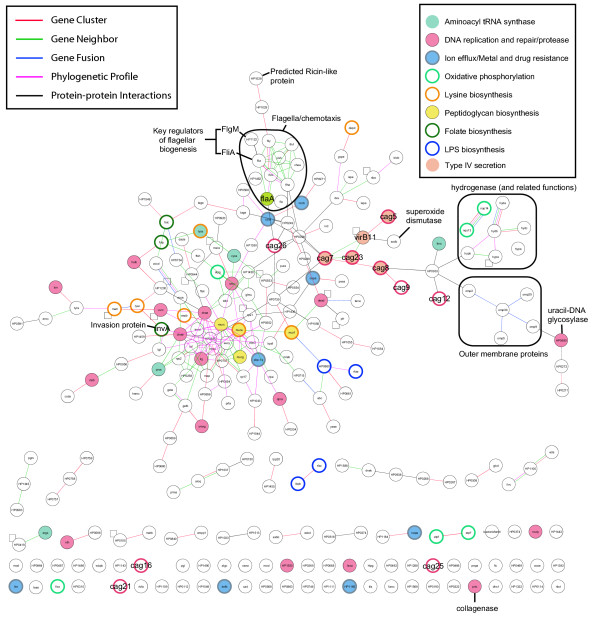
**Annotated prolinks network view of 263 genes identified to beputatively functionally associated with one or more of the 26 cytotoxin-associated *cag *genes in *H. pylori***. This filtered network was obtained through selection of genes in biclusters of putatively co-regulated containing one or more *cag *gene(s). The *cag *genes are indicated with pink node borders. See inset keys for description of node and edge coloring.

Altogether 71 proteins associated with the *cag *gene biclusters were of unknown function. Among these unknown function proteins are four conserved secreted proteins including one protein (HP0028) linked through protein-protein interactions to Cag26 (CagA) – a key pathogenesis protein [33]. Another set of interesting unknown function proteins were HP1028 and HP1029 connected via gene cluster (operon) edges to FliY and FliM – key flagellar switch proteins. Also present in this operon is an alternate sigma factor (σ^28^) gene *fliA *which has been implicated in mediating transcription of FlaA (also present in the flagellar gene association network (Fig 3)), the major flagellar subunit required for both motility [34] as well as gastric colonization [35]. Note that co-expression analysis alone was not sufficient to find these relationships. Moreover, both functional associations and protein-protein interactions are notoriously noisy; however, our use of a combined analysis of all of these orthogonal data sources increases the likelihood that these relationships are real.

To further explore putative functions of these key genes of unknown function, we retrieved their protein sequences (by broadcasting the genes to the STRING goose). The protein sequences were manually submitted to Robetta, a structure prediction server [36]. Among the various proteins analyzed the most striking was the match of predicted three dimensional structure of HP1028 to B-chain of ebulin (PDB: 1 hwm), a ricin-like toxin. Proteins with the conserved ricin domain are ribosome inactivating proteins widely distributed in plants, fungi, algae and bacteria. This putative function for HP1028, coupled to its putative functional association with flagellar proteins, implicates it in a likelyrole in *H. pylori *pathogenesis. In a similar manner, future functional exploration of additional unknown function genes in our candidate set (Additional file 1) will provide basis for discovery of potentially new candidate genes involved in pathogenesis of *H. pylori*.

This case study illustrates the exploration of one set of heterogeneous data, using one particular combination of web resources and gaggled programs. The flexibility of the Gaggle enables any other kinds of exploration, combining other kinds of data, employing other analytical programs and web resources, and using different analytical styles (emphasizing genomics, or statistics, or simulation). In other words, a user can choose her/his style of data analysis through extensive trial and error operations using the Gaggle to layout a landscape of complex diverse data from which to tease out biological insights.

#### Targeted users of the Gaggle

Through the example above we illustrate how the Gaggle is designed to serve biologists at all points along the spectrum, from biologists who conduct most of their analyses using spreadsheets and web browsers to statistically savvy computational biologists who can write their own R code. However, note that, with the exception of the R goose, all current programs in the Gaggle are point-and-click applications, and fully useful to the non-programming biologist. Among these point-and click applications, are applications such as TIGR MeV [12], which provide the biologist quick access to a suite of statistical analysis tools. More importantly, although TIGR MeV development will continue independent of the Gaggle, users of the Gaggle will benefit from advances in this third party tool. This exemplifies the benefit of coupling existing popular open source software. As and when more popular software are developed we will make them part of the Gaggle.

With the addition of the R goose, a new class of biological work is supported, through which even the most proficient R programmer may benefit from a collection of point-and-click geese, for instance, for the visual display of STRING associations, KEGG pathways, and Cytoscape networks, all with just a few mouse clicks.

In our experience, there is yet a third class of biologists, who have no prior experience with R; but who use the Gaggle to explore their data with the point-and-click geese; and are also not opposed to using a few simple one-line R commands as long as they have a cribsheet to work from. We provide this cribsheet on the Gaggle website and intend to populate it with useful commands that are clearly described from a biologist's standpoint. Some of the commands in the cribsheet tell the user how to filter their data, normalize it, and find intersection and/or union between two matrices or gene lists.

Thus, the Gaggle provides a setting in which point-and-click exploration may be gently expanded to include the sort of statistical data exploration, which is becoming indispensable in analyzing complex systems biology data. In other words the Gaggle can be (and is currently) used both by novices to computational biology and also by high end statisticians familiar with R. thereby improving communication among collaborators of diverse expertise.

## Future Work

In addition to the straightforward task of adding new geese to the Gaggle (for example, a goose for Gene Ontology annotation and for Robetta structure prediction), we also wish to add new capabilities to existing geese. For example, we plan to add simple scripting capabilities to the Boss, probably using Jython, to support 'goose pipelines', in which the result from one goose may be automatically sent to another. Another ambitious goal currently planned is to add a unified 'save state' capability to the Gaggle, requiring (primarily) some extensions to each participating goose.

## Conclusion

The Gaggle is a minimal, effective and open-ended system for integrating software and data sources used in systems biology analyses. The Gaggle's effectiveness comes from the recognition that four simple data messages each free of biological semantics, and a judicious use of the Java programming language, are all that is needed to integrate diverse types of data and software. More importantly, the Gaggle is easily extensible and new software and databases can be easily converted into geese of the Gaggle with little effort. This has advantage over other approaches which require tight coupling of software and databases and therefore extensive effort to integrate new resources into the framework. This we emphasize is an important consideration because many valuable databases and software already exist and new resources are constantly emerging -if we are to take full advantage of all these existing and forthcoming resources without reformatting data or extensively reconfiguring those resources, we predict that a strategy such as the Gaggle will prove to be invaluable.

## Source code and Gaggle availability

All of the Gaggle source code, and all of the geese mentioned in this manuscript, are available, with full documentation along with a growing number of ready-to-use "Gaggles" of model organisms on the Gaggle website.

## Authors' contributions

**PS **Conceived and initiated the project. Developed and implemented the method and the resultant computer program. Wrote the manuscript.

**DJR **Obtained and parsed out the relevant biological conditions in the *H. pylori *microarray data. Allowed access to pre-publication results of the *cMonkey *algorithm.

**RB **Obtained and parsed out the relevant biological conditions in the *H. pylori *microarray data. Allowed access to pre-publication results of the *cMonkey *algorithm.

**NSB **Conceived and initiated the project. Provided direction, feedback on the quality of results, software design and crafted the case study. Wrote the manuscript.
